# Human papilloma and other DNA virus infections of the cervix: A population based comparative study among tribal and general population in India

**DOI:** 10.1371/journal.pone.0219173

**Published:** 2019-06-27

**Authors:** Supriti Ghosh, Ranjitha S. Shetty, Sanjay M. Pattanshetty, Sneha D. Mallya, Deeksha Pandey, Shama Prasada Kabekkodu, Veena G. Kamath, Navya Prabhu, Joslin D’souza, Kapaettu Satyamoorthy

**Affiliations:** 1 Department of Cell and Molecular Biology, School of Life Sciences, Manipal Academy of Higher Education, Manipal, Karnataka, India; 2 Department of Community Medicine, Kasturba Medical College, Manipal Academy of Higher Education, Manipal, Karnataka, India; 3 Centre for Indigenous Population, Manipal Academy of Higher Education, Manipal, Karnataka, India; 4 Prasanna School of Public Health, Manipal Academy of Higher Education, Manipal, Karnataka, India; 5 Department of Obstetrics and Gynaecology, Kasturba Medical College, Manipal Academy of Higher Education, Manipal, Karnataka, India; Yenepoya Medical College, Yenepoya University, INDIA

## Abstract

**Background:**

Despite being preventable, cervical cancer remains a major health concern among women. Persistent Human Papillomavirus (HPV) and other viral co-infections may influence cervical dysplasia. We determined and compared the prevalence and risk factors of cervical viral infections among the tribal and general population of southern coastal Karnataka, India.

**Methods:**

A population-based cross-sectional survey was conducted among 1140 and 1100 women from tribal and general population, respectively. Cervical infections with HPV, Epstein-Barr Virus (EBV), Cytomegalovirus (CMV) and Herpes-Simplex Virus (HSV) were examined using polymerase chain reactions (PCR) and DNA sequencing.

**Results:**

HPV prevalence was higher among tribal women (40.6%) than general population (14.3%) while the prevalence of EBV (55.1%) and CMV (49.4%) were lower among tribal women than general population (74.3% and 77.5%, respectively). HSV infection was observed in tribal women only (1.8%). Among HR-HPV strains, HPV-18 was predominant among tribal population (28.3%) while, HPV-16 was predominant among the general population (9.1%). Infections were associated with age, educational status, unemployment and personal hygiene of tribal women. Phylogenetic analysis revealed that HPV-16 variants of tribal participants were closely related to non-European sublineages indicating greater risk of HPV persistence and carcinogenesis.

**Conclusion:**

The study provides a comparative estimate for DNA virus infections of the cervix among women from general as well as tribal population in this region and also reveals a different type-specific pattern of viral infection. Further research is required to delineate the role of specific interactions between multiple virus infections and their role in carcinogenesis.

## Introduction

Cervical cancer is the leading cancer among women aged 15–44 years, especially in the developing countries, with human papillomavirus (HPV) infection being the key etiological factor [1]. At any given time, about 5% of the women in the general population in India harbour HPV-16/18 in the cervix [2]. In spite of being an important risk factor, HPV infection alone may be insufficient to cause cervical cancer. Infection with HIV, other opportunistic pathogens and interaction with host genetic and epigenetic factors may contribute to cervical carcinogenesis [3]. Globally, viral coinfections account for about 10–15% of all cancers, of which more than 85% occur in low and middle-income countries [4]. Moreover, the members of the herpesviridae family including Epstein-Barr virus (EBV), Cytomegalovirus (CMV) and Herpes-Simplex virus (HSV) have been known to establish lifelong latency in the hosts and implicated to increase the risks of cervical neoplasia [5,6]. Despite the advances made to delineate the association of viral co-infection with cancer, the primary infection levels of viruses in the normal and asymptomatic individuals remains to be elucidated.

The ethnic tribes form an integral part of India’s social diversity constituting 8.6% of the total population [7]. High prevalence of sexually transmitted infections (STIs) is observed among tribal women due to their distinct customs, making them vulnerable to cervical cancer [8]. Hence, the pattern of cervical viral infections among tribal women needs to be determined. There is also a paucity of such data among the general population in this region. Therefore, a survey was carried out to estimate the prevalence of HPV, EBV, CMV and HSV, co-infections and risk factors in these populations.

## Materials and methods

### Ethical statement

Permission was obtained from the State Tribal Welfare Department (STWD), Karnataka, Integrated Tribal Development Project (ITDP) and District Health Authority to carry out the study in the district. The study proposal was approved by the Institutional Ethics Committee, Kasturba Hospital, Kasturba Medical College, Manipal, prior to initiation of the study (Registration no.: ECR/146/Inst/KA/2013; Project Approval no.: IEC– 181/2013 and IEC-23/2017). Married women aged 20–65 years were included after obtaining a written consent from the eligible participants willing to take part in the study.

### Study population and study design

A population-based cross-sectional survey was carried out among women from the tribal communities (Koraga, Marathi Naika, and Malekudiya—in accordance with The Scheduled Castes and Scheduled Tribes Orders (Amendment) Act, 1976 and as inserted by Act 39 of 1991) and from the general population of Udupi district situated in south-coastal Karnataka, of southern India. About 41,613 tribal people inhabit Udupi district (Koraga, Marathi Naika and Malekudiya tribes being 11,133, 28,524 and 1956, respectively). Among the three communities, Koraga is primitive and original inhabitants of coastal Karnataka. Traditional occupation of this community is basket weaving and rope making with overall low socio-economic status as illiteracy, unemployment and alcohol and tobacco use prevail in these communities.

Considering the HPV prevalence to be 16.9% among rural women reported earlier [9] for a precision of 15%, with 95% confidence interval and a non-response rate of 20%, the sample size was estimated to be 1102 for each population. In the tribal population, women were recruited from three communities based on probability proportional to size (PPS) method. Eligible women excluding those who were pregnant or lactating at the time of survey or who had undergone hysterectomy/previously diagnosed with and/or treated for cervical lesions and those with uterine prolapse were included in the study.

### Data and sample collection strategy

**Fig 1:**
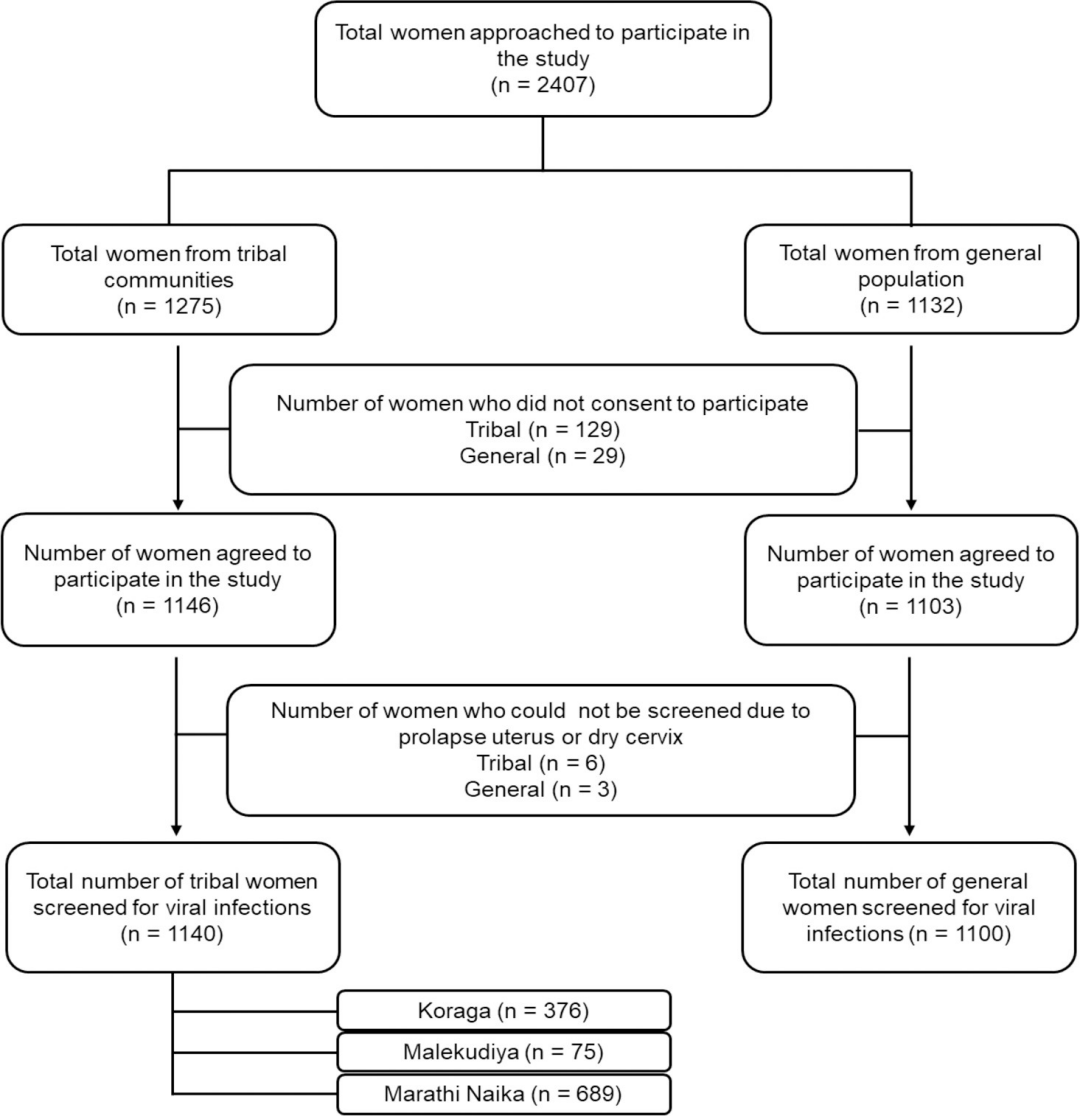
Participant recruitment. Flowchart summarizing the enrolment of participants from tribal communities and general population for the study.

Socio-demographic, gynaecologic and reproductive data were collected using pre-designed questionnaire, after obtaining written informed consent. Socio-economic status was assessed using modified Udai Pareek scale [10]. Participants were requested to attend screening camps on pre-informed dates, organized at nearby primary health centres (Fig 1).

On the day of camp, exfoliated cervical samples were obtained by a trained medical doctor /a trained nurse with the help of a cytobrush and transported to the laboratory in sterile 15ml tubes containing Phosphate Buffered Saline (PBS) solution on ice. Samples were centrifuged and pellets were stored at -20°C till further processing.

### DNA isolation

The exfoliated cervical cells were digested with RNaseA (10mg/ml) and proteinase K (10mg/ml), treated with 1M Tris (pH 7.4), 0.5M EDTA and 20% SDS and incubated overnight at 37°C. DNA was extracted by standard phenol-chloroform method followed by precipitation with ethanol, dissolved in sterile Milli-Q water and stored at -20°C until further use.

### Virus identification and genotyping

Viral type-specific PCRs were performed to detect the presence of HPV, EBV, CMV and HSV, as described in the literature [11–14]. HPV subtypes were identified by sequencing of L1 locus as published earlier in 3130 Genetic Analyzer using Big Dye Terminator kit according to manufacturer’s instructions (ThermoFisher, USA) [11]. The sequences were aligned against the viral genome using PaVE (Papillomavirus Episteme) and NCBI BLAST algorithms.

### Statistical and bioinformatic analysis

Data was analysed using Statistical Package for Social Sciences (SPSS) version 16.0. Categorical data was summarized as mean ±standard deviation (SD). Univariable and multivariable logistic regressions were performed to estimate the crude and adjusted odds ratios (OR) with corresponding 95% confidence intervals (CI). The factors showing an association with viral infections at a p <0.2 on univariable analysis were included in multivariable analysis.

Software R and online tool HighCharts were used for construction of clusters, heatmaps and graphs.

### Phylogenetic tree construction

DNA sequences of HPV-16 L1 locus from our results were used for construction of phylogenetic tree by maximum parsimony approach using MEGA package 7.0 with 1000 bootstrap replicates. The sequences were aligned against references collected from GenBank which included NC_001526.3 and 10 sublineages of HPV-16 viz., A1 (K02718.1), A2 (AF536179.1), A3 (HQ 644236.1), A4 (AF534061.1), B1 (AF536180.1), B2 (HQ64429.1), C (AF472509.1), D1 (HQ644257.1), D2 (HQ644270.1) and D3 (AF402678.1).

## Results

### Socio-demographic, reproductive and gynaecological characteristics

**Fig 2:**
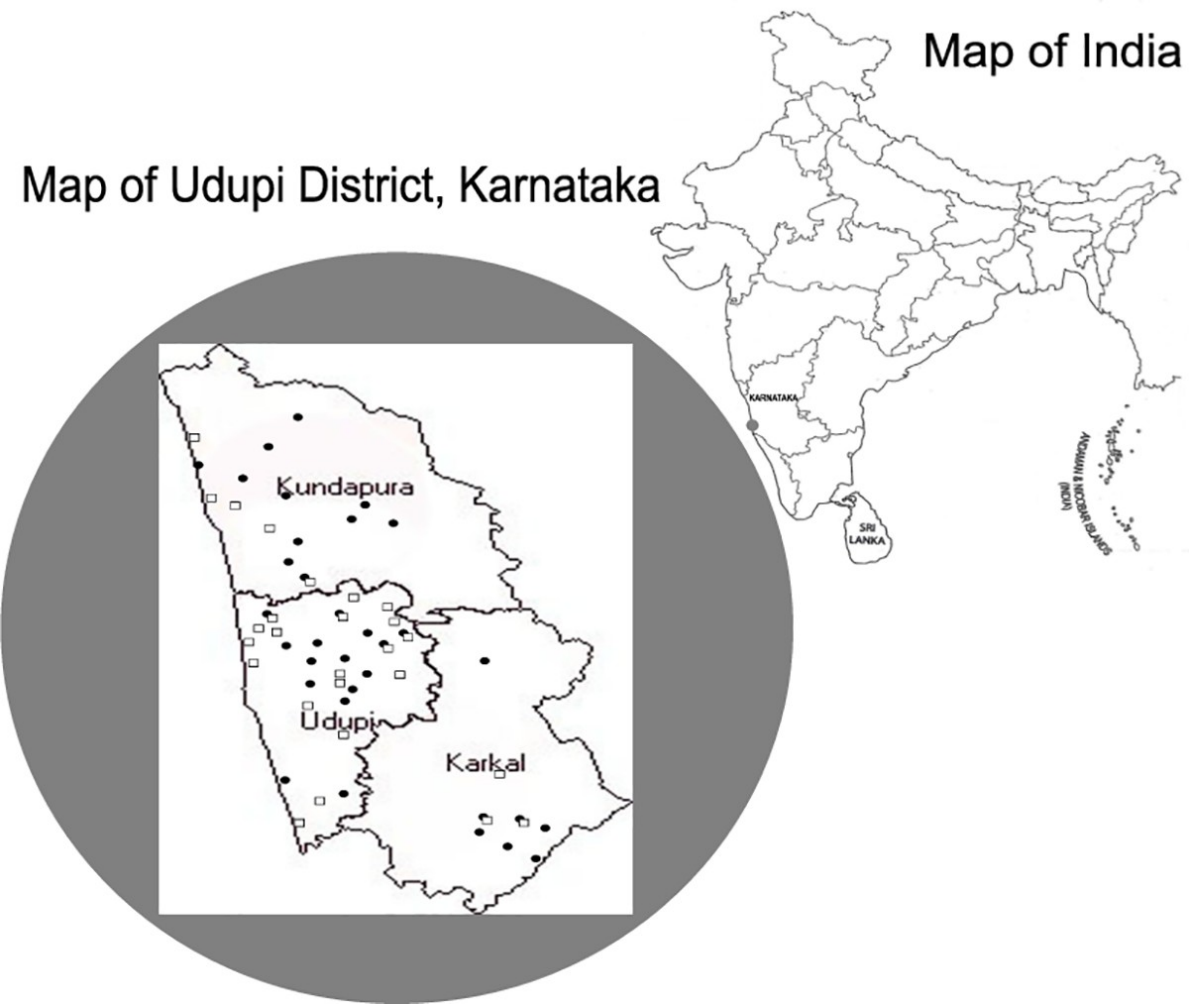
Location of screening camps. Map illustrating the geographical location of the screening camps across three regions of Udupi district of southern coastal Karnataka, India, which has an area of 3880 square kilometres. Black dots represent the camps organised for tribal population and unfilled squares represent the camps organised for general population.

A total of 1140 tribal women and 1100 from general population were screened through 80 camps (Fig 2). Demographic, reproductive and gynaecological characteristics of the study populations were collected (Table 1). Of the 1140 tribal women screened, the highest proportion of women were from Marathi Naika community (60.4%) followed by Koraga (33.0%) and Malekudiya (6.6%). Mean age of participants from tribal and general population were 40 (±10.1) and 43 (±9.2) years, respectively. Mean age at menarche were 13 (±1.3) years and 14 (±1.7) years and menopause were 45 (±5.0) years and 46 (±5.2) years among tribal and general population, respectively. Mean age at marriage was 21 (±3.8) years and 23 (±4.3) years among tribal and general population, respectively.

**Table 1:** Demographics, reproductive and gynaecological characteristics of the study populations.

Variables	Frequency (%)	
	Tribal population	General population
	(n = 1140)	(n = 1100)
**Age group (in years)**		
≤30	259 (22.7)	94 (8.5)
31–45	572 (50.2)	594 (54.0)
>46	309 (27.1)	412 (37.5)
**Marital status**		
Married	1018 (89.3)	987 (89.7)
Widowed	114 (10.0)	105 (9.5)
Separate	8 (0.7)	8 (0.8)
**Educational level**		
<5 years	654 (57.4)	331 (30.1)
≥5 years	486 (42.6)	769 (69.9)
**Employment status**		
Employed	509 (44.6)	500 (45.5)
Home-maker	631 (55.4)	600 (54.5)
**Socio-economic status**		
Low	697 (61.1)	409 (37.2)
Medium	443 (38.9)	691 (62.8)
**Smokeless tobacco consumption**		
Ever	280 (24.6)	140 (12.7)
Never	860 (75.4)	960 (87.2)
**Age at marriage (in years)**		
≤18	318 (27.9)	191 (17.4)
19–24	595 (52.2)	573 (52.1)
>24	22 (19.9)	336 (30.5)
**Parity**		
Nulliparous	68 (6.0)	59 (5.4)
1–4	977 (85.7)	990 (90.0)
>4	95 (8.3)	51 (4.6)
**History of abortion**		
Present	161 (14.1)	255 (23.2)
Absent	979 (85.9)	845 (76.8)
**Married more than once**		
Yes	9 (0.8)	4 (0.4)
No	1131 (99.2)	1096 (99.6)
**Menstrual cycle**		
Regular	1046 (91.8)	1049 (95.4)
Irregular	94 (8.2)	51 (4.6)
**Attained menopause**		
Yes	319 (28.0)	379 (34.5)
No	821 (72.0)	721 (65.5)
**Type of sanitary napkin used**		
Home-made	981 (86.1)	708 (64.4)
Disposable	159 (13.9)	392 (35.6)
**Gynaecological complaint**		
White discharge	331 (29.0)	453 (41.2)
Post-coital bleeding	2 (0.2)	9 (0.8)
Severe lower back ache	885 (77.6)	757 (68.8)
Genital lesions	10 (0.9)	1 (0.1)
Dyspareunia	8 (0.7)	7 (0.6)

### Prevalence of viral infections

A distinct pattern of viral infections was observed in the cervical samples of both populations. The prevalence of overall HPV infection was 40.6% and 14.3% among tribal women and general population, respectively, i.e. higher among the tribal women (OR = 4.27; 95% CI 2.14–8.52). In contrast, prevalence of EBV (55.1% vs. 74.2%) and CMV (49.5% vs. 77.5%) infections were lower among tribal population (OR = 0.43; 95% CI 0.24–0.78 and OR = 0.28; 95% CI 0.15–0.52, respectively). Prevalence of HSV infection among tribal women was 1.8% and absent in women from general population. Infections with at least one virus was observed in approximately 82% of the tribal women and 91.5% of the women from the general population. Interestingly, infection with HPV, independent of viral co-infection, was detected in only 8.8% and 0.5% of tribal and general population, respectively.

Among HPV negative tribal women, the prevalence of EBV, CMV and HSV infection was 42.4%, 56.0% and 1.9%, respectively, whereas the prevalence of EBV and CMV infections in HPV negative women from general population, was 74.3% and 76.1%, respectively. The prevalence of these viral infections was compared among HPV positive and HPV negative women from both populations (S1 Fig). Prevalence of CMV infection among HPV negative tribal women was higher compared to HPV positive women (OR = 3.93; 95% CI 2.20–7.06) while prevalence of EBV infection was higher among HPV positive than HPV negative tribal women (OR = 1.91; 95% CI 1.08–3.29).

### Distribution of HPV subtypes

**Fig 3:**
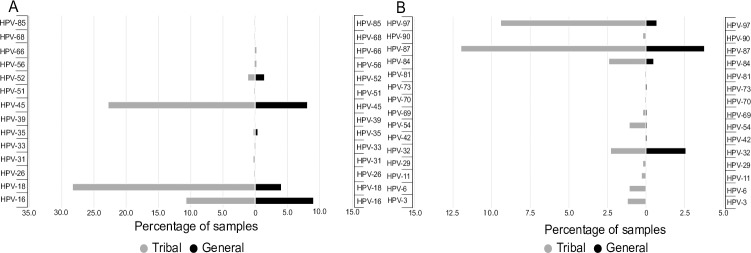
Distribution of HPV subtypes among women from tribal and general population. A) Distribution of high risk (HR) HPV subtypes among the two study populations. B) Distribution of low risk (LR) HPV subtypes among the two study populations.

A total of 14 high-risk (HR) and 15 low-risk (LR) HPV subtypes were detected from both the groups (Fig 3). Interestingly, HPV-18 infection was higher among tribal while HPV-16 was predominant among general population. The prevalent HR-HPV subtypes among tribal women were HPV-18 (28.3%), HPV-45 (22.8%) and HPV-16 (10.7%) whereas, those among general population were HPV-16 (9.1%), HPV-45 (8.1%) and HPV-18 (4.1%) (Fig 3A). The predominant LR-HPV subtype among both the populations was HPV-87 (12.0% and 3.8%, respectively) (Fig 3B). Other HR- and LR-HPV subtypes were present in a small proportion of both the groups.

Infection with single HPV subtype was observed in 14.4% and 5.1% while, infections with multiple HPV subtypes was observed in 24.7% and 8.6% of tribal and general population, respectively. The prevalent single HPV infection among tribal women was HPV-18 (8.1%), followed by HPV-16 (4.9%) and HPV-6 (<1%) while that among general population was HPV-16 (4.1%) followed by HPV-18 (<1%).

### Pattern of multiple HPV infections

**Fig 4:**
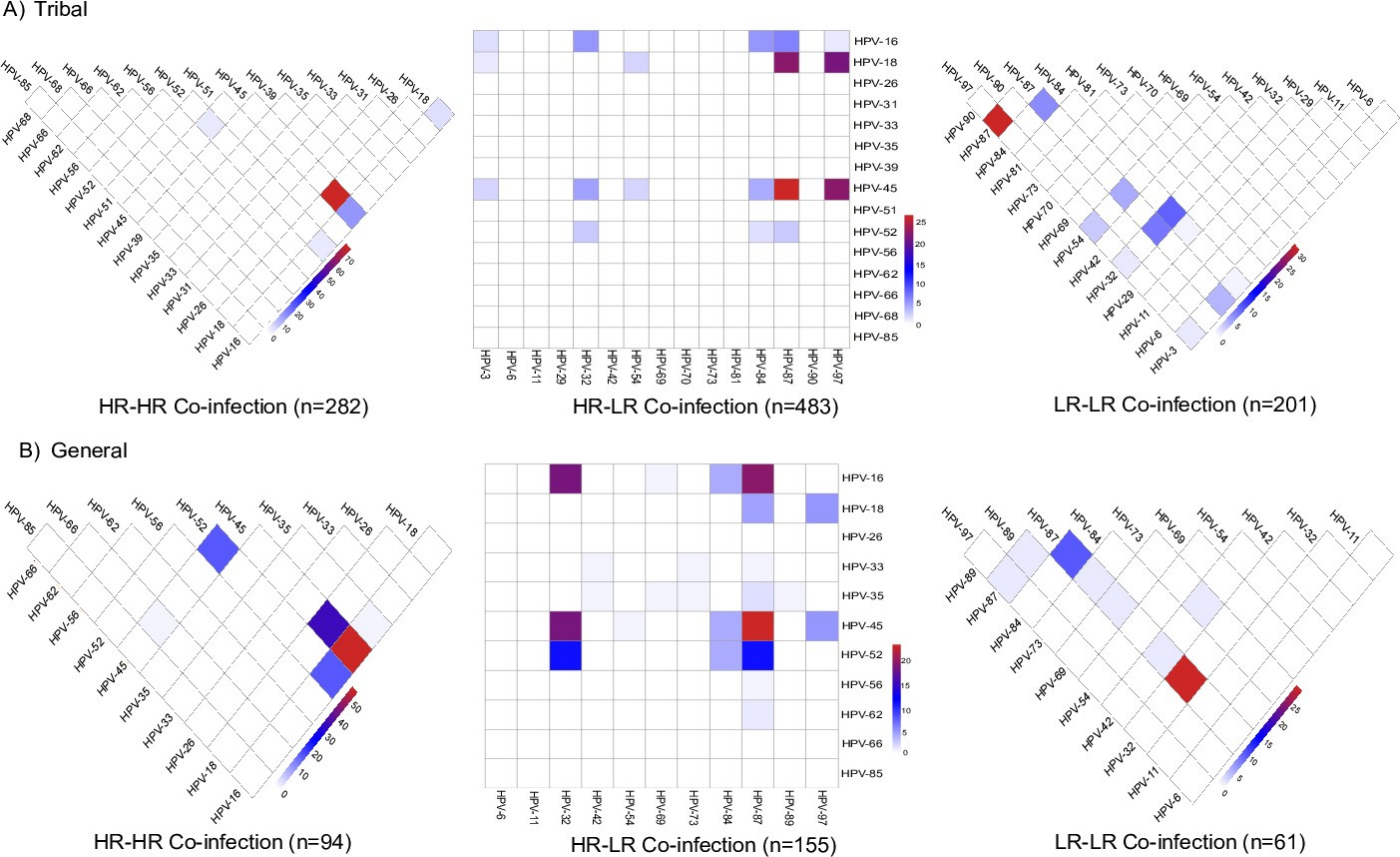
Multiple HPV infections in the samples. Pattern of co-infections of HPV subtypes in samples infected with multiple HPV subtypes. A) Pattern of coinfection of HR-HR, HR-LR and LR-LR HPV subtypes among the tribal population. B) Pattern of coinfection of HR-HR, HR-LR and LR-LR HPV subtypes among the tribal population. HR: high risk; LR: low risk HPV subtypes.

Among the HPV positive women, about 60% were infected with multiple HPV subtypes in both populations. Pattern of infections with multiple HPV subtypes was evaluated among tribal and general population (Fig 4A and 4B, respectively). In the HR-HR HPV group, the predominant co-infection among tribal population were HPV-18 and 45 (77.7%) followed by HPV-16 and 45 (16.3%), HPV-16 and 18 (5.3%) and HPV-16 and 52 (4.6%). Among the general population, co-infection of HPV-16 and 45 was predominant (55.3%) followed by HPV-18 and 45 (36.2%). In the HR-LR infection category, HPV-45 and 87 co-infection was observed in 26.9% and 23.9% of tribal and general population, respectively. Among tribal population, in the LR-LR infection category, co-infection of HPV-87 and 97 was predominant (31.3%) while, in general population, co-infection of HPV-32 and 87 was predominant (29.5%).

### Pattern of viral co-infections

The occurrence and pattern of cervical viral co-infections was evaluated in the samples (S2 Fig). Presence of viral co-infections among tribal population (49.8%) was lower compared to general population (65.6%) (OR = 0.47; 95% CI 0.25–0.88). Of the 568 co-infected women from tribal population, co-infection of EBV+CMV was present in 34.9%, HPV+EBV in 30.5% and HPV+CMV in 3.3% of samples while, 28.9% showed HPV+EBV+CMV triple infection. Co-infection of all four viruses was present in 0.4% of women. Among the 722 co-infected women from general population, 78.9% demonstrated dual infection of EBV+CMV, 5.1% with HPV+CMV and 2.4% with HPV+EBV and 13.6% HPV+EBV+CMV triple infection.

Samples exhibiting co-infection with HPV were investigated for the distribution of co-infections with HR- and LR-HPV subtypes (S1 Table). Viral co-infections with HR-HPV subtypes were predominant with a cumulative percentage of 59.7% in tribal and 29.9% in general population. Among HPV negative samples, frequent co-infection was observed were EBV+CMV; 29.4% in tribal and 60.4% in general population while <1% co-infection with EBV+HSV, CMV+HSV and EBV+CMV+HSV was observed among HPV negative tribal samples.

### Association of viral infections with the risk factors

**Fig 5:**
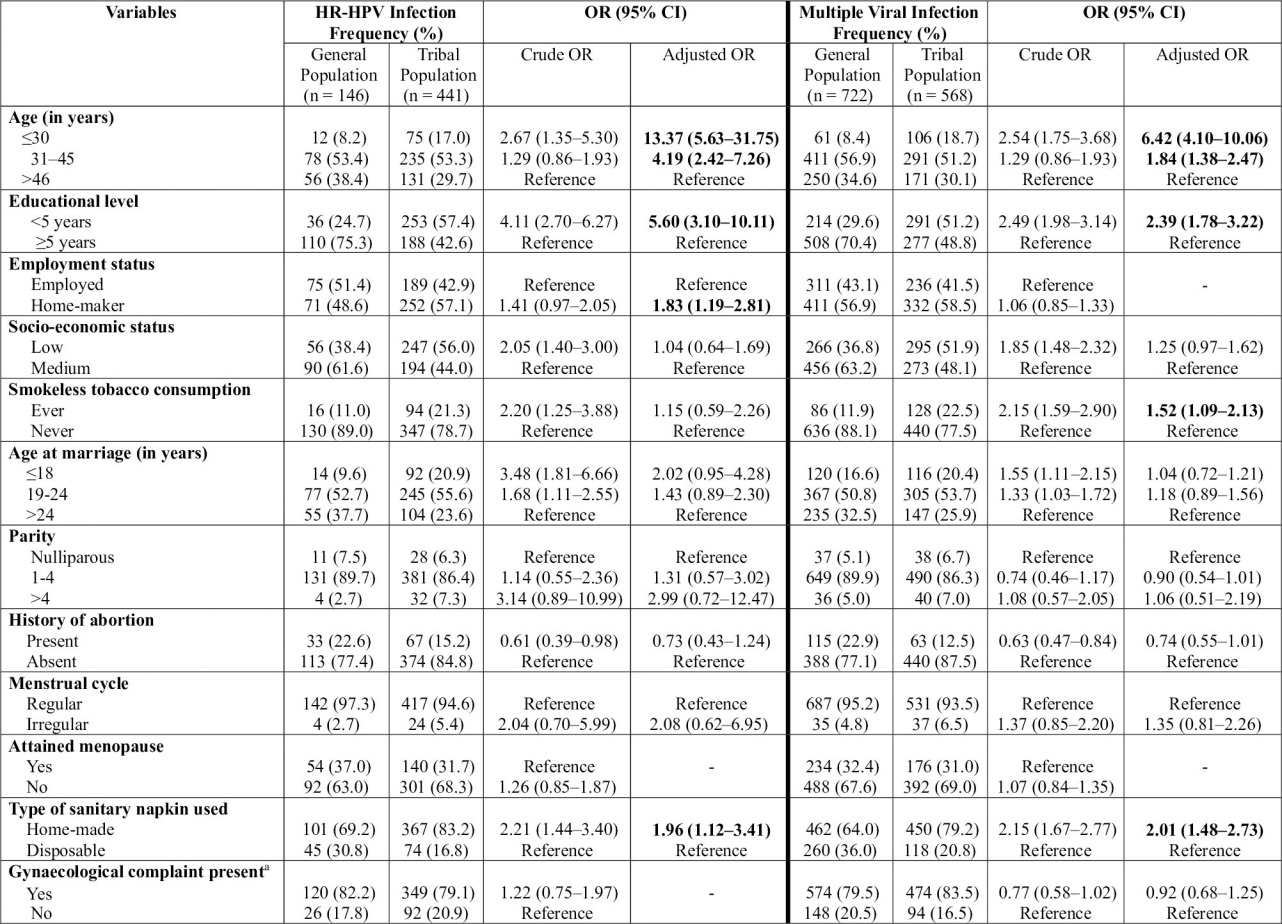
Association of viral infections with risk factors. Table depicting the association of demographics, sexual and reproductive characteristics with presence of HR-HPV and multiple viral infections in the study populations. Bold indicates statistical significance based on 95% confidence interval. ^a^Includes discharge per vagina, severe lower back ache, post-coital bleeding, history of genital lesions, and dyspareunia.

The univariable and multivariable associations between variables and presence of HR-HPV and multiple viral infections were determined (Fig 5). Of 1140 women from tribal and 1100 women from general population, 38.7% and 13.3% women, respectively, presented infection with HR-HPV subtypes. Multivariable analysis mutually adjusted for risk factors with p<0.2 indicated that tribal women who were younger in age; ≤30 years (OR = 13.37; 95% CI 5.63–31.75) and 31 to 45 years (OR = 4.19; 95% CI 2.42–7.26), had low education level (OR = 5.60; 95% CI 3.10–10.11), were home-makers (OR = 1.83; 95% CI 1.19–2.81) and used home-made sanitary napkins (OR = 1.96; 95% CI 1.12, 3.41) had higher HR-HPV infection than general population.

About 65.6% women from general and 49.8% from tribal population showed multiple viral infections. Following multivariable analysis, factors associated with multiple viral infections among tribal women included young age of the participants; ≤30 years (OR = 6.42; 95% CI 4.10–10.06) and 31–45 years (OR = 1.84; 95% CI 1.38–2.47), having <5 years of education (OR = 2.39; 95% CI 1.78–3.22), positive history of smokeless tobacco consumption (OR = 1.52; 95% CI 1.09–2.13), and using home-made sanitary napkins (OR = 2.01; 95% CI 1.48–2.73).

Similarly, associations of variables and presence of EBV and CMV infections in all the women (S2 Table) and such associations with presence of EBV and CMV infections among HPV negative women from both the populations (S3 Table) were determined. Association of risk factors indicates similar correlations among tribal women as indicated above.

### Phylogenetic analysis of HPV-16 L1 variants

**Fig 6:**
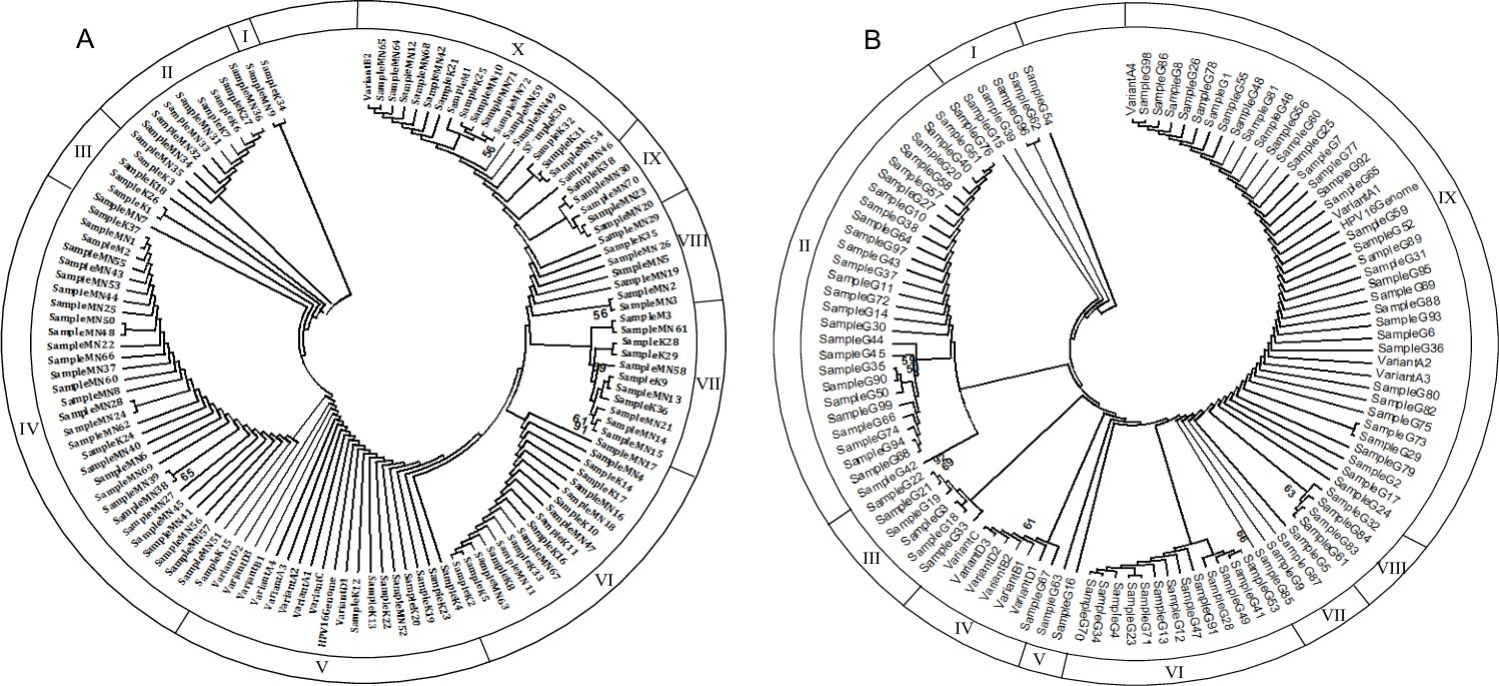
Phylogenetic analysis of HPV-16 sample variants based on L1 locus. A) Phylogenetic tree constructed from HPV-16 variants from tribal population. B) Phylogenetic tree constructed from HPV-16 variants from general population. Maximum Parsimony method was used to construct the phylogenetic trees by Mega package 7.0. The standard sequences include GeneBank accession no. NC_00156.3 and HPV-16 sublineages A1 (K02718.1), A2 (AF536179.1), A3 (HQ644236.1), A4 (AF534061.1), B1 (AF536180.1), B2 (HQ64298.1), D1 (HQ644257.1), D2 (HQ644270.1) and D3 (AF402678.1). The numbers closest to the branch points are bootstrap values (1000 replicates). Values lower than 50% are not shown. G: General population; K: Koraga; M: Malekudiya; MN: Marathi Naika.

Maximum parsimony trees of HPV-16 L1 were constructed from molecular phylogenetic analysis of 11 reference sequences aligned with 113 tribal and 99 general population sample variants with 1000 bootstrap replicates to test the robustness of the tree (Fig 6). Phylogenetic tree with tribal samples demonstrated 10 clusters (I to X) with 1.8%, 7.9%, 4.4%, 27.4%, 7.1%, 9.7%, 6.2%, 4.4% and 15.9% samples, respectively (Fig 6A). Among these 7.1% samples aligned to North American sublineage-D1 and 0.8% aligned to African-2 sublineage-B2. Phylogenetic tree with samples from general population demonstrated 9 clusters (I to IX) with 5.1%, 28.3%, 6.1%, 1.0%, 2.0%, 13.1%, 4.0%, 4.0% and 36.4% samples, respectively (Fig 6B). Among these, 26.3% samples aligned with European sublineages-A1 (16.2%) and A2 (10.1%) and only 1.0% sample aligned with Asian sublineage-A4.

## Discussion

In this study, higher prevalence of HPV (40.6% vs. 14.3%) and HSV (1.8% vs. none) and lower prevalence of EBV (55.1% vs. 74.2%) and CMV (49.5% vs. 77.5%) were observed in tribal population than general population. Prevalence of HPV among general population was in concordance with that observed in asymptomatic women from British Columbia (12.3%), Iran (13%), Taiwan (13.8%) and rural Mexico (14.7%) [15–18]. Studies from rural Thailand (6.3%), Egypt (10.4%) and Pakistan (4.74%) reported lower HPV prevalence while studies from Brazil (24.5%) and Hawaii (25.6%) reported higher HPV prevalence than that of general population in our study [19–23]. A study involving women with normal cervical cytology in Serbia reported similar HPV prevalence (41.3%) as the tribal population of our study [24]. Previous studies conducted among rural women of Southern India, middle-aged women of Maharashtra and pre-adolescent, adolescent and young tribal girls of three Indian states reported the overall HPV prevalence to be from 10.3% to 16.9%, which are less compared to the HPV prevalence observed among tribal women in our study [9,25,26]. Earlier reports from this region indicated HPV prevalence of 20% to 82.5% across non-malignant, pre-malignant and cancer samples, 57.7% among HIV positive women with cervical abnormalities while HPV was detected in <1% urine samples [27–29]. A recent study involving rural and ethnic women in China revealed similar difference in HPV prevalence among two populations [30]. This and other studies [19,24,25,30,31] report HPV-16 as the predominant subtype which is in agreement with that of general population of our study; although HPV-18 (8.1%) was more prevalent in our tribal population. Data from our study and available literature including meta-analyses, suggest differential pattern of prevalence and distribution of HPV and its subtypes vary depending on ethnicity and geography [32,33]. In our study about 60% of the HPV positive samples from both the populations demonstrated infections with multiple HPV subtypes. The prevalence of multiple HPV infections among normal samples ranges from 2.6–20.2% while such infections are reported to be higher in case of cervical abnormalities and lesions [15,18,24,34]. In a recent study conducted among 100 women with cervical abnormality, 68% of the samples demonstrated multiple HPV infections [35]. The HPV subtypes, therefore, may exhibit both cooperative and competitive interactions during cervical carcinogenesis [36] which needs to be validated using molecular studies.

EBV and CMV infections were lower among tribal women than women from general population. A few studies have suggested the prevalence of EBV and CMV in the cervical secretions from healthy women and it ranges from 10–30% [37]. This is in concordance with a study conducted in Andhra Pradesh, India, that reported the prevalence of EBV and CMV to be 20% and 26%, respectively, in cervical shedding among healthy women [38]. However, this range is much lower than that observed in our study. Additionally, the seroprevalence of EBV and CMV in the blood of healthy individuals differ greatly from their prevalence in the cervix. For instance, EBV infection in adults have been linked to nasopharyngeal carcinoma, posttransplant lymphoproliferative diseases (PTLDs), Hodgkin’s lymphoma and gastric carcinoma and the seroprevalence of EBV among healthy individuals is reported to be over 90% globally [39]. Similarly, epidemiology studies from different parts of India suggest the seropositivity of CMV IgG antibody to be about 80–90% among women of childbearing age [40]. While the CMV IgG seropositivity was 93.2% among healthy blood donors in Ghana, a recent study conducted in Germany reported the seroprevalence of CMV to be 62.3% among healthy adult women [41,42]. This indicates that detection of serum antibodies in the blood give a systemic and generalized perspective than use of cervical cells for detection of viral genes which provides a more localized view. Comparison of EBV and CMV infections in the presence and absence of HPV in the study populations revealed that EBV infection was higher among HPV positive and CMV infection was higher among HPV negative tribal women. Presence of EBV positive HPV negative cervical carcinoma with expression of EBV proteins have been indicative of EBV as a co-factor for cervical carcinogenesis [43]. Moreover, frequency of CMV positivity in cervical shedding has been linked to cervical lesions indicating the possible involvement of CMV in oncogenesis [44]. The high prevalence of EBV and CMV infection irrespective of HPV infection may be an indicative of a primary, latent infection by these viruses rendering the host susceptible to subsequent HPV infections.

High prevalence of cervical viral co-infections was observed among tribal (49.8%) and general (65.3%) population with EBV and CMV co-infection being predominant, irrespective of HPV status. Studies have reported co-infection of HPV and EBV in non-malignant and malignant cervical tissues and its significant association with cervical carcinogenesis [45]. EBV has been reported to transform EBV/C3d receptor bearing cervical cells thereby increasing their receptiveness to oncogenic stimuli [37]. Cervical CMV infections have also been observed in cervical biopsies as well as young women with HPV infections attending STD clinics [46,47]. The immediate early genes of CMV transactivate other viral and host genes, thereby increasing risk of carcinogenesis [5]. Presence of co-infections of HPV with EBV and CMV, and their synergistic effect on cervical oncogenesis need to be explored.

Cervical HPV infections are reported to be acquired around adolescence, peak at middle-age and declining after 45 years, across the globe [48]. In concordance with this, our data showed similar trend of infections with age, wherein tribal women who were younger had higher HR-HPV and multiple viral infections compared to their general population counterparts. Similarly, tribal women with low education status, unemployed, consuming smoke-less forms of tobacco and using home-made sanitary napkins showed higher HR-HPV infections than general population. These variables can be considered as surrogate markers for overall low socio-economic status which in turn is strong predictor of HPV infection and can be reflected in both sexual and non-sexual factors including hygiene and nutritional deficiencies [49].

HPV-16 L1 variants exhibit diverse geographical distribution and pathogenicity. The non-European sublineages, especially Asian (A4) sublineage, has been reported to be involved with persistent HPV infection, development of cervical lesions and progression towards high grade squamous intraepithelial lesions (HSIL) [50,51]. In our study, 7.9% of the HPV-16 sample variants from tribal population aligned to non-European sublineages, viz. 7.1% North American and 0.8% African-2 sublineage. Whereas, among general population samples, 26.3% aligned with European and 1.0% with non-European (Asian) sublineages. This might be an indicative of higher risk of persistent HPV infection and oncogenic progression among the tribal population compared to the general population.

This community-based study is first of its kind in this region assessing prevalence of cervical HPV and other DNA viral infections, co-infections and their risk factors among tribal and general populations. Higher prevalence of HPV and other viral single and co-infections was observed in both the populations. Study also highlights some of the socio-demographic and reproductive characteristics as potential risk factors of cervical HR-HPV and multiple viral infections among tribal women. The prevalent infections with herpesviruses, including EBV and CMV among non-malignant samples irrespective of HPV infection status may suggest a synergistic infection. We, therefore, recommend molecular testing of DNA viruses together with Pap smear tests for identification of latent viral infections at early stages and follow-up cohort studies to identify their role in causing cervical abnormalities. This study provides baseline data on HPV and mixed viral infections among the women from two socio-culturally diverse groups. Longitudinal studies to explore the role of multiple virus infections in cervical carcinogenesis is the need of the hour as it would help in policy making and introduction of population-specific interventions against HR-HPV and multiple viral infections.

## Supporting information

S1 Fig Other viral infections among HPV positive and negative women. Comparison of prevalence of EBV, CMV and HSV infections among HPV positive and HPV negative women from tribal and general population (HPV positive tribal n = 463, HPV positive general n = 157, HPV negative tribal n = 677 and HPV negative general n = 943). *: p-Value <0.05; **: p-Value <0.01; ***: p-value <0.001.
(TIF)

S2 Fig Pattern of viral co-infection. Pattern of co-infection of different viruses among the samples infected with more than one virus, i.e. samples with multiple viral infections (n = 568 tribal, 722 general).
(TIF)

S1 Table Distribution of viral co-infections with HR- and LR- HPV subtypes among the study populations. (DOCX)

S2 Table Association of demographic, sexual and reproductive characteristics with presence of EBV and CMV infections among both the populations. (DOCX)

S3 Table Association of demographic, sexual and reproductive characteristics with presence of EBV and CMV infections among HPV negative samples from both the populations. (DOCX)
