# Supplementary figures and images for: Human papilloma and other DNA virus infections of the cervix: A population based comparative study among tribal and general population in India
Source: PLoS One. 2019 Jun 27;14(6):e0219173. doi: 10.1371/journal.pone.0219173 (PMC6597196; doi:10.1371/journal.pone.0219173)

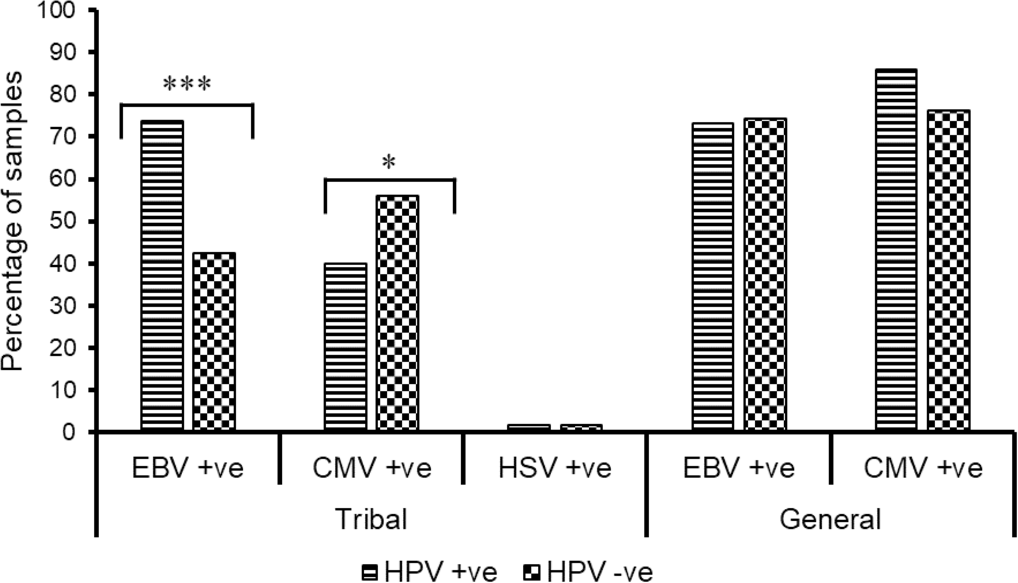

Supplement: S1 Fig — Comparison of prevalence of EBV, CMV and HSV infections among HPV positive and HPV negative women from tribal and general population (HPV positive tribal n = 463, HPV positive general n = 157, HPV negative tribal n = 677 and HPV negative general n = 943). *: p-Value <0.05; **: p-Value <0.01; ***: p-value <0.001. (TIF) [file pone.0219173.s001.tif]

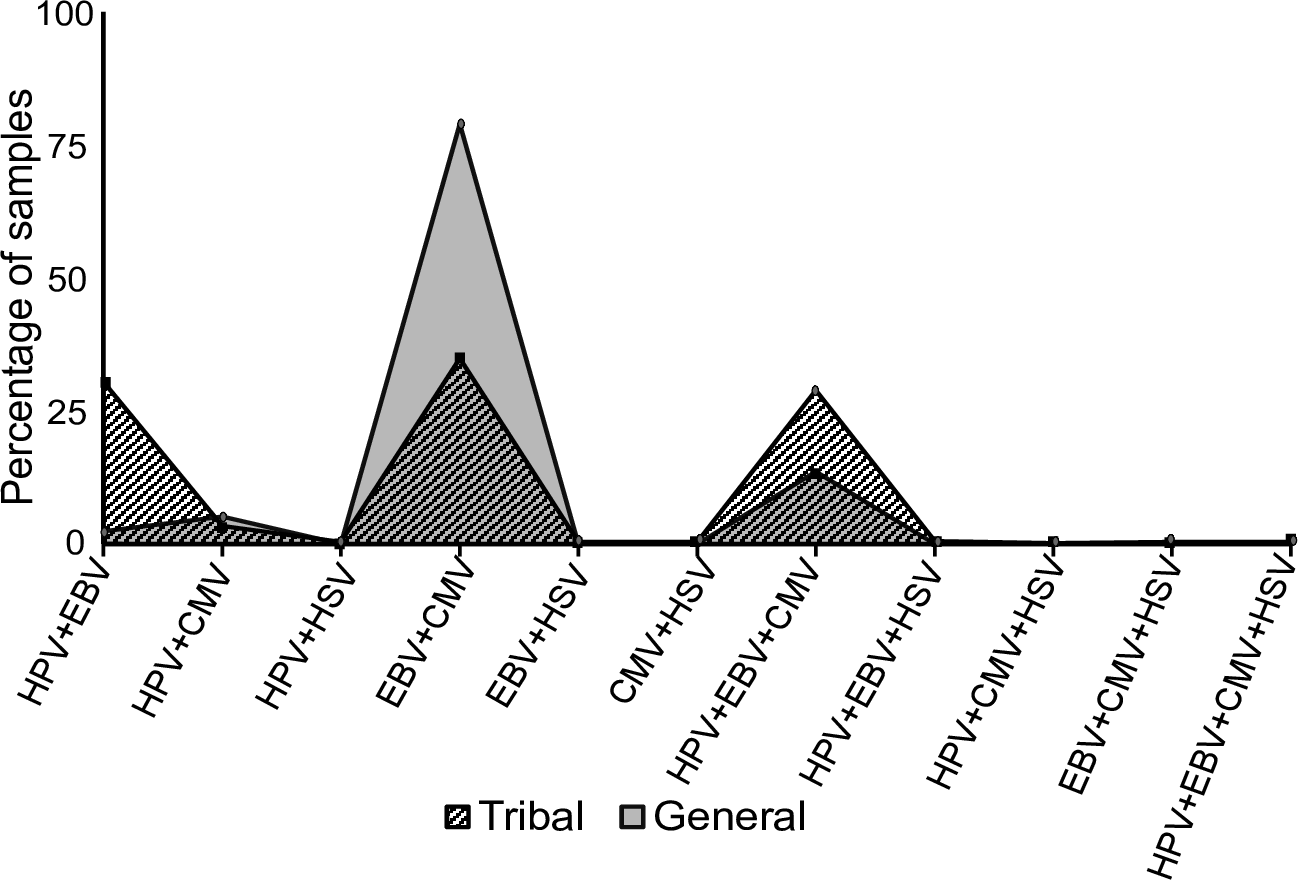

Supplement: S2 Fig — Pattern of co-infection of different viruses among the samples infected with more than one virus, i.e. samples with multiple viral infections (n = 568 tribal, 722 general). (TIF) [file pone.0219173.s002.tif]
