# Supplementary material for: Human papilloma and other DNA virus infections of the cervix: A population based comparative study among tribal and general population in India
Source: PLoS One. 2019 Jun 27;14(6):e0219173. doi: 10.1371/journal.pone.0219173 (PMC6597196; doi:10.1371/journal.pone.0219173)
Supplement: S1 Table — (DOCX) [file pone.0219173.s003.docx]

**S1 table.** **Distribution of viral co-infections with HR- and LR- HPV subtypes among the study populations.**

| **Viral Co-infection** | **Frequency (%)** | |
| --- | --- | --- |
|  | **Tribal**  **(n = 568)** | **General**  **(n = 722)** |
| HR-HPV+EBV | 164 (28.9) | 14 (1.9) |
| HR-HPV+CMV | 19 (3.3) | 37 (5.1) |
| HR-HPV+HSV | 1 (0.2) | 0 |
| HR-HPV+EBV+CMV | 149 (26.2) | 93 (12.9) |
| HR-HPV+EBV+HSV | 3 (0.5) | 0 |
| HR-HPV+CMV+HSV | 1 (0.2) | 0 |
| HR-HPV+EBV+CMV+HSV | 2 (0.4) | 0 |
| LR-HPV+EBV | 99 (17.4) | 5 (0.7) |
| LR-HPV+CMV | 7 (1.2) | 18 (2.5) |
| LR-HPV+HSV | 1 (0.2) | 0 |
| LR-HPV+EBV+CMV | 70 (12.3) | 34 (4.7) |
| LR-HPV+EBV+HSV | 1 (0.2) | 0 |
