# Supplementary material for: Human papilloma and other DNA virus infections of the cervix: A population based comparative study among tribal and general population in India
Source: PLoS One. 2019 Jun 27;14(6):e0219173. doi: 10.1371/journal.pone.0219173 (PMC6597196; doi:10.1371/journal.pone.0219173)
Supplement: S2 Table — (DOCX) [file pone.0219173.s004.docx]

**S2 Table.** **Association of demographic, sexual and reproductive characteristics with presence of EBV and CMV infections among both the populations.**

| **Variables** | **EBV Infection**  **Frequency (%)** | | **OR (95% CI)** | | **CMV Infection**  **Frequency (%)** | | **OR (95% CI)** | |
| --- | --- | --- | --- | --- | --- | --- | --- | --- |
|  | General Population  (n = 816) | Tribal Population  (n = 628) | Crude OR | Adjusted OR | General Population  (n = 853) | Tribal Population  (n = 564) | Crude OR | Adjusted OR |
| **Age (in years)**  ≤30  31–45  >46 | 68 (8.3)  467 (57.2)  281 (34.4) | 111 (17.7)  318 (50.6)  199 (31.7) | 2.31 (1.62–3.28)  0.96 (0.76–1.21)  Reference | **6.39 (4.14–9.84)**  **1.93 (1.45– 2.56)**  Reference | 69 (8.1)  474 (55.6)  310 (36.3) | 130 (23.0)  284 (50.4)  150 (26.6) | 3.89 (2.74–5.53)  1.24 (0.97–1.58)  Reference | **8.09 (4.89–13.38)**  **2.07 (1.43–3.01)**  Reference |
| **Educational level**  <5 years  ≥5 years | 242 (29.7)  574 (70.3) | 328 (52.2)  300 (47.8) | 2.59 (2.09–3.22)  Reference | **2.88 (2.15–3.86)**  Reference | 255 (29.9)  598 (70.1) | 279 (49.5)  285 (50.5) | 2.30 (1.84–2.86)  Reference | **2.65 (1.95–3.59)**  Reference |
| **Employment status**  Employed  Home-maker | 356 (43.6)  460 (56.4) | 257 (40.9)  371 (59.1) | Reference  1.12 (0.91–1.38) | - | 374 (43.8)  479 (56.2) | 242 (42.9)  322 (57.1) | Reference  1.04 (0.84–1.29) | - |
| **Socio-economic status**  Low  Medium | 296 (36.3)  520 (63.7) | 325 (51.8)  303 (48.2) | 1.88 (1.53–2.33)  Reference | 1.24 (0.97–1.59)  Reference | 324 (38.0)  529 (62.0) | 321 (56.9)  243 (43.1) | 2.16 (1.74–2.68)  Reference | **1.42 (1.09–1.84)**  Reference |
| **Smokeless tobacco consumption**  Ever  Never | 101 (12.4)  715 (87.6) | 142 (22.6)  486 (77.4) | 2.07 (1.56–2.74)  Reference | 1.35 (0.97–1.87)  Reference | 104 (12.2)  749 (87.8) | 135 (23.9)  429 (76.1) | 2.27 (1.71–3.00)  Reference | **1.76 (1.26–2.47)**  Reference |
| **Age at marriage (in years)**  ≤18  19-24  >24 | 132 (16.2)  425 (52.1)  259 (31.7) | 129 (20.5)  339 (54.0)  160 (25.5) | 1.58 (1.16–2.16)  1.29 (1.01–1.65)  Reference | 0.95 (0.66–1.36)  1.04 (0.79–1.36)  Reference | 154 (18.1)  432 (50.6)  267 (31.3) | 144 (25.5)  292 (51.8)  128 (22.7) | 1.95 (1.43–2.66)  1.41 (1.09–1.83)  Reference | 1.04 (0.72–1.51)  1.20 (0.90–1.60)  Reference |
| **Parity**  Nulliparous  1-4  >4 | 44 (5.4)  734 (90.0)  38 (4.7) | 41 (6.5)  539 (85.8)  48 (7.6) | Reference  0.79 (0.51–1.22)  1.36 (0.74–2.47) | - | 45 (5.3)  764 (89.6)  44 (5.2) | 38 (6.7)  484 (85.8)  42 (7.4) | Reference  0.75 (0.48–1.17)  1.13 (0.62–2.07) | - |
| **History of abortion**  Present  Absent | 189 (23.2)  627 (76.8) | 94 (15.0)  534 (85.0) | 0.58 (0.44–0.77)  Reference | **0.69 (0.51–0.93)**  Reference | 192 (22.5)  661 (77.5) | 79 (14.0)  485 (86.0) | 0.56 (0.42–0.75)  Reference | 0.73 (0.53–1.01)  Reference |
| **Menstrual cycle**  Regular  Irregular | 771 (94.5)  45 (5.5) | 585 (93.2)  43 (6.8) | Reference  1.26 (0.82–1.94) | - | 818 (95.9)  35 (4.1) | 518 (91.8)  46 (8.2) | Reference  2.08 (1.32–3.27) | Reference  **1.79 (1.05–3.04)** |
| **Attained menopause**  Yes  No | 265 (32.5)  551 (67.5) | 197 (31.4)  431 (68.6) | Reference  1.05 (0.84–1.32) | - | 286 (33.5)  567 (66.5) | 158 (28.0)  406 (72.0) | Reference  1.30 (1.03–1.64) | Reference  1.44 (0.98–2.10) |
| **Type of sanitary napkin used**  Home-made  Disposable | 551 (62.6)  305 (37.4) | 497 (79.1)  131 (20.9) | 2.26 (1.78–2.88)  Reference | **2.32 (1.73–3.11)**  Reference | 557 (65.3)  296 (34.7) | 469 (83.2)  95 (16.8) | 2.62 (2.02–3.41)  Reference | **3.02 (2.20–4.13)**  Reference |
| **Gynaecological complaint present**^a^  Yes  No | 566 (76.0)  179 (24.0) | 523 (83.3)  105 (16.7) | 1.58 (1.20–2.06)  Reference | 1.34 (1.00–1.79)  Reference | 612 (78.2)  171 (21.8) | 481 (85.3)  83 (14.7) | 1.62 (1.21–2.16)  Reference | 1.32 (0.96–1.82)  Reference |

Bold indicates statistical significance based on the 95% confidence interval.

^a^Includes discharge per vagina, severe lower back ache, post-coital bleeding, history of genital lesions and dyspareunia.
