# Supplementary material for: Human papilloma and other DNA virus infections of the cervix: A population based comparative study among tribal and general population in India
Source: PLoS One. 2019 Jun 27;14(6):e0219173. doi: 10.1371/journal.pone.0219173 (PMC6597196; doi:10.1371/journal.pone.0219173)
Supplement: S3 Table — (DOCX) [file pone.0219173.s005.docx]

**S3 table. Association of demographic, sexual and reproductive characteristics with presence of EBV and CMV infections among HPV negative samples from both the populations.**

| **Variables** | **EBV+ HPV-**  **Frequency (%)** | | **OR (95% CI)** | | **CMV+ HPV-**  **Frequency (%)** | | **OR (95% CI)** | |
| --- | --- | --- | --- | --- | --- | --- | --- | --- |
|  | General Population  (n = 701) | Tribal Population  (n = 287) | Crude OR | Adjusted OR | General Population  (n = 718) | Tribal Population  (n = 379) | Crude OR | Adjusted OR |
| Age, in years  ≤30  31–45  >46 | 58 (8.3)  399 (56.9)  244 (34.8) | 52 (18.1)  138 (48.1)  97 (33.8) | 2.26 (1.45–3.51)  0.87 (0.64–1.18)  Reference | **5.20 (3.02–8.93)**  **1.61 (1.11–2.32)**  Reference | 59 (8.2)  399 (55.6)  260 (36.2) | 96 (25.3)  179 (47.2)  104 (27.4) | 4.07 (2.74–6.04)  1.12 (0.84–1.50)  Reference | **8.68 (4.84–15.59)**  **1.97 (1.27–3.05)**  Reference |
| Educational level  <5 years  ≥5 years | 216 (30.8)  485 (69.2) | 140 (48.8)  147 (51.2) | 2.14 (1.61–2.83)  Reference | **2.25 (1.55–3.26)**  Reference | 224 (31.2)  494 (68.8) | 191 (50.4)  188 (49.6) | 2.24 (1.74–2.89)  Reference | **2.37 (1.66–3.37)**  Reference |
| Employment status  Employed  Home-maker | 302 (43.1)  399 (56.9) | 107 (37.3)  180 (62.7) | Reference  1.27 (0.96–1.69) | Reference  **1.42 (1.04–1.95)** | 307 (42.8)  411 (57.2) | 164 (43.3)  215 (56.7) | Reference  0.98 (0.76–1.26) | - |
| Socio-economic status  Low  Medium | 254 (36.2)  447 (63.8) | 145 (50.5)  142 (49.5) | 1.80 (1.36–2.37)  Reference | **1.38 (1.004–1.90)**  Reference | 272 (37.9)  446 (62.1) | 232 (61.2)  147 (38.8) | 2.59 (2.00–3.34)  Reference | **1.75 (1.29–2.38)**  Reference |
| Smokeless tobacco consumption  Ever  Never | 91 (13.0)  610 (87.0) | 70 (24.4)  217 (75.6) | 2.16 (1.53–3.06)  Reference | **1.66 (1.11–2.49)**  Reference | 89 (12.4)  629 (87.6) | 93 (24.5)  286 (75.5) | 2.30 (1.67–3.17)  Reference | **1.83 (1.24–2.69)**  Reference |
| Age at marriage, in years  ≤18  19-24  >24 | 120 (17.1)  364 (51.9)  217 (31.0) | 65 (22.6)  149 (51.9)  73 (25.4) | 1.61 (1.08–2.41)  1.22 (0.88–1.69)  Reference | 0.98 (0.63–1.55)  0.93 (0.65–1.33)  Reference | 142 (19.8)  362 (50.4)  214 (29.8) | 109 (28.8)  195 (51.5)  75 (19.8) | 2.19 (1.52–3.15)  1.54 (1.12–2.11)  Reference | 1.14 (0.74–1.75)  1.24 (0.87–1.77)  Reference |
| Parity  Nulliparous  1-4  >4 | 34 (4.9)  632 (90.2)  35 (5.0) | 16 (5.6)  245 (85.4)  26 (9.1) | Reference  0.82 (0.45–1.52)  1.58 (0.72–3.45) | - | 35 (4.9)  642 (89.4)  41 (5.7) | 20 (5.3)  327 (86.3)  32 (8.4) | Reference  0.89 (0.51–1.57)  1.37 (0.67–2.80) | - |
| History of abortion  Present  Absent | 160 (22.8)  541 (77.2) | 45 (15.7)  242 (84.3) | 0.63 (0.44–0.91)  Reference | 0.72 (0.49–1.06)  Reference | 162 (22.6)  556 (77.4) | 52 (13.7)  327 (86.3) | 0.55 (0.39–0.77)  Reference | 0.74 (0.50–1.09)  Reference |
| Menstrual cycle  Regular  Irregular | 661 (94.3)  40 (5.7) | 264 (92.0)  23 (8.0) | Reference  1.44 (0.85–2.45) | Reference  1.54 (0.86–2.77) | 688 (95.8)  30 (4.2) | 345 (91.0)  34 (9.0) | Reference  2.26 (1.36–3.76) | Reference  **2.18 (1.18–4.03)** |
| Attained menopause  Yes  No | 227 (32.4)  474 (67.6) | 91 (31.7)  196 (68.3) | Reference  1.03 (0.77–1.39) | - | 238 (33.1)  480 (66.9) | 111 (29.3)  268 (70.7) | Reference  1.20 (0.91–1.57) | Reference  1.27 (0.82–1.99) |
| Type of sanitary napkin used  Home-made  Disposable | 437 (62.3)  264 (37.7) | 219 (76.3)  68 (23.7) | 1.95 (1.42–2.66)  Reference | **1.92 (1.33–2.79)**  Reference | 465 (64.8)  253 (35.2) | 322 (85.0)  57 (15.0) | 3.07 (2.23–4.24)  Reference | **3.26 (2.23–4.77)**  Reference |
| Gynaecological complaint present^a^  Yes  No | 475 (74.8)  160 (25.2) | 241 (84.0)  46 (16.0) | 1.77 (1.23–2.54)  Reference | **1.56 (1.06–2.29)**  Reference | 507 (77.6)  146 (22.4) | 329 (86.8)  50 (13.2) | 1.90 (1.34–2.69)  Reference | **1.53 (1.04–2.27)**  Reference |

Bold indicates statistical significance based on the 95% confidence interval.

^a^Includes discharge per vagina, severe lower back ache, post-coital bleeding, history of genital lesions and dyspareunia.
